# Effective or Harmful—Evaluation of Locally Applied Antibiotics on Adipose Tissue during Lipofilling to the Breast—An In Vitro Study

**DOI:** 10.3390/ijms24032323

**Published:** 2023-01-24

**Authors:** Yannick F. Diehm, Emre Gazyakan, Yiping Wang, Laura C. Siegwart, Valentin Haug, Dimitra Kotsougiani-Fischer, Ulrich Kneser, Sebastian Fischer

**Affiliations:** 1 Department of Hand-, Plastic and Reconstructive Surgery, Burn Center, BG Trauma Center Ludwigshafen, Heidelberg University, Ludwig-Guttmann-Strasse 13, 67071 Ludwigshafen, Germany; 2 AESTHETIKON Plastische Chirurgie Mannheim & Heidelberg, L9 8, 68161 Mannheim, Germany

**Keywords:** lipofilling, breast reconstruction, breast cancer, postoperative infection, antibiotics

## Abstract

Lipofilling is a frequently used and safe procedure for breast reconstruction. One of the most feared complications is soft tissue infection following lipofilling. Because of this, some surgeons propose the practice of rinsing fat grafts with antibiotics. This study investigates the effect of antibiotic rinses on fat grafts in an in vitro model. Adipocytes and stem cells were isolated from fat tissue harvested during 24 lipofilling procedures and incubated with different doses of clindamycin or cefazolin. Cell viability, metabolism, proliferation, and differentiation capacities were analyzed by gross morphology, fluorescence staining, -(4,5-Dimethylthiazol-2-yl)-2,5-diphenyltetrazoliumbromid (MTT-), and Glyceraldehyde 3 Phosphate Dehydrogenase (G3PD)-assay as well as reactive oxygen species (ROS)-assay. Cefazolin and clindamycin led to significant reduction of cell viability of adipocytes. High doses of both antibiotics led to a rupture of adipocytes with visible free lipid droplets. Cell metabolism was significantly decreased after incubation with both antibiotics. There was a significant increase in ROS production. Exposure to clindamycin and cefazolin led to morphological changes in stem cells in a dose- and time-dependent manner. Furthermore, differentiation potential was significantly reduced. Antibiotic susceptibility testing, however, showed that low concentrations of antibiotics effectively inhibited bacterial growth in contaminated fat grafts. This study confirms that rinsing fat grafts with clindamycin or cefazolin not only overly prevents infection but also has cytotoxic and metabolic effects on adipocytes. Therefore, based on these results, the routine clinical application in high doses cannot be recommended.

## 1. Introduction

Breast cancer is the most common malignancy among women worldwide with a still growing incidence [1,2,3]. Although cancer development is not fully understood, age, intake of oral contraceptives, hormone substitution as well as genetic predisposition have been identified as significant risk factors. The term breast cancer summarizes more than 20 different tumor entities with varying courses of disease and prognoses [4]. Depending on the histologic growth pattern, the two most frequent types are ductal or lobular carcinomas. With a frequency of 10–15% of all breast cancer types, triple-negative breast cancer is characterized by a lack of expression of the markers estrogen and progesterone receptors as well as HER2 [4]. This type is associated with the lowest survival and highest metastasis rate among breast cancer patients. Fortunately, an improved treatment regimen led to increasing survival rates after adequate breast cancer treatment, and quality of life and cosmetic outcomes of breast cancer survivors become increasingly important [5]. Implant-based or autologous breast reconstruction are frequently performed procedures within the breast cancer treatment regimen. However, minor additional interventions are often needed to achieve satisfactory results. Lipofilling is a commonly used technique for breast augmentation and correction of breast contour deformities after breast cancer treatment [6,7,8]. Additionally, lipofilling is a widely used technique in breast surgery, e.g., cosmetic breast augmentation and correction of breast deformities such as tuberous breasts. A study by Gentile compared outcomes of implant-based breast augmentation with autologous fat grafting and found that the latter resulted in more natural results, yet, with a lower retention rate of breast volume [9]. By the addition of adipose-derived stem cells (ADSCs) these volume retention rates could be further boosted in clinical application [8]. In regard to tuberous breasts, a comparison between fat grafting an mastopexy combined with breast implants showed excellent cosmetic results with minimal scarring after two fat grafting procedures [7]. Besides the apparent beneficial effects of lipofilling on breast contour and volume, fat grafting has been associated with reduction in post-operative pain, improved skin quality, and angiogenesis [10,11]. However, the variable fat absorption and limited amount of fat that can be transferred often warrant multiple lipofilling procedures [9,12].

Severe postoperative infections are the most common and serious complications following lipofilling, occurring in 3–5% of all lipofilling procedures [13,14]. Leading pathogens are staphylococcus aureus or coagulase-negative staphylococcus [15]. Perioperative antimicrobial prophylaxis is performed by many surgeons prior to transplantation by rinsing fat grafts with antibiotic solutions. The antibiotics used as prophylaxis are mainly clindamycin or cefazolin. Yet, there is scarce evidence on either the effectiveness or the potential antibiotic-induced harmful alterations on fat tissue. In particular, the effect of antibiotics on ADSCs, important cells for the promotion of fat graft survival and regeneration, is still widely unknown. Mesenchymal stem cells, such as ADSCs, are multipotent adult stem cells able to differentiate into cells of ectodermal, endodermal, and mesenchymal origin [16]. ADSCs inherit a great regenerative potential mediated by their anti-inflammatory and immune-suppressive effects as well as their capabilities to directly differentiate into different cell types [16]. In terms of fat grafting, the ADSCs induce neovascularization by secreting large amounts of vascular endothelial growth factor (VEGF), hepatocyte growth factor and basic fibroblast growth factors and thus mediating fat graft survival [17,18].

The purpose of this study was to investigate the potential harmful effects of clindamycin and cefazolin on adipocytes and adipose-derived stem cells and to define a safe but effective concentration range of antibiotic solutions for fat graft rinsing prior to transplantation.

## 2. Results

### 2.1. Stem Cell Characterization

After ADSCs isolation, stem cells displayed a fibroblast-like morphology during expansion. Adipogenic and osteogenic differentiation of ADSCs led to cells being positively stained for Oil Red O and Alizarin Red. Flow cytometry showed that cultivated ADSC displayed CD29, CD73 and CD90 while omitting CD31 and CD45.

### 2.2. Gross Morphology

**Figure 1:**
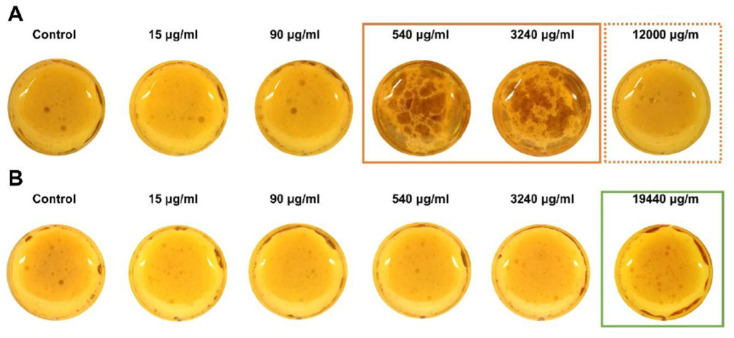
Gross morphology of adipocytes. (**A**) Adipocytes treated with clindamycin. The orange frame marks the appearance of ruptured adipocytes for group AB3 and AB4. The dashed frame indicates the paradox effect of the highest clindamycin dose with intact adipocytes. (**B**) Adipocytes treated with cefazolin. The green frame marks ruptured adipocytes in group AB5.

Clindamycin incubation led to a large amount of free lipid droplets being released from ruptured adipocytes at a concentration of 540 μg/mL and 3240 μg/mL compared to the control group (Figure 1). However, adipocytes appeared to be intact without floating lipid droplets at the highest concentration of 12,000 μg/mL. For the cefazolin groups, rupturing of adipocytes with the appearance of free lipid droplets appeared at the highest concentration of 19,440 μg/mL.

### 2.3. Hoechst/Propidium Iodide (PI) Fluorescence Staining

**Figure 2:**
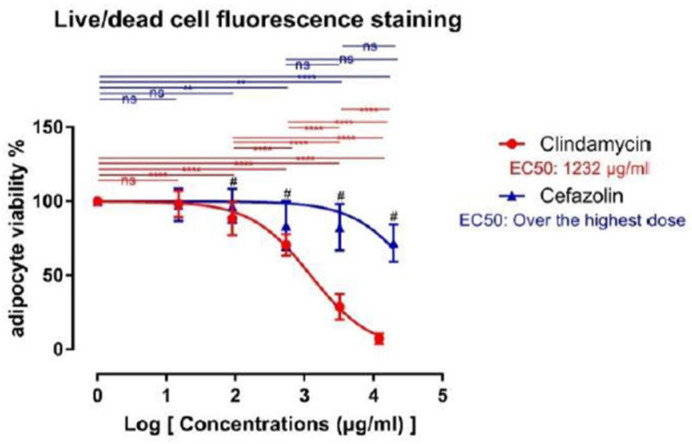
Sigmoidal dose-response curve for live/dead staining. Data are shown as mean ± standard deviation. Asterix denotes statistically significant difference vs. the control group (ns = not significant; ** = *p* < 0.01; **** = *p* < 0.0001). Brackets indicate statistically significant differences between corresponding groups of clindamycin and cefazolin treated adipocytes (# = *p* < 0.05). ns means non-significant. EC50 characterizes the concentration leading to a 50% reduction.

15 μg/mL of clindamycin did not lead to any significant differences in the occurrence of dead cells compared to the control. However, live/dead cell analysis showed that a concentration of 90 μg/mL already leads to a statistically significant decrease of vital adipocytes (71.92 ± 43 vs. 3.38 ± 7.50%, *p* < 0.0001). Clindamycin incubation showed a dose-dependent effect on adipocyte viability. With increasing clindamycin concentrations, the number of dead adipocytes increased while the occurrence of healthy adipocytes decreasing, respectively. These effects were statistically significant between all study groups and the control (Control: 71.92 ± 4.63 vs. AB3: 50.65 ± 4.46%, AB4: 20.49 ± 5.48%, and AB5: 5.34 ± 2.74%, *p* < 0.0001). The inter-group comparison showed statistically significant differences between groups AB2, AB3, AB4, and AB5, respectively (Figure 2).

Inhibitory effects of cefazolin were not as strong as for clindamycin. The lower concentrations of cefazolin (15 and 90 μg/mL) did not lead to a significant reduction in adipocyte viability in our experiments. Higher cefazolin concentrations (540, 3240 and 19,440 μg/mL), however, significantly reduced the survival rates of adipocytes, again in a dose-dependent manner (control: 71.49 ± 5.54 vs. AB3: 59.36 ± 9.64%, and AB4: 58.72 ± 10.73%, *p* < 0.01; vs. AB5 cefazolin: 50.9 ± 6.52, *p* < 0.0001). Between groups AB3, AB4, and AB5, no statistically significant differences were observed. When we compared clindamycin with cefazolin groups, we found a significant stronger inhibition of adipocyte viability for groups AB2, AB3, AB4, and AB5 when treated with clindamycin.

### 2.4. XTT Assay

**Figure 3:**
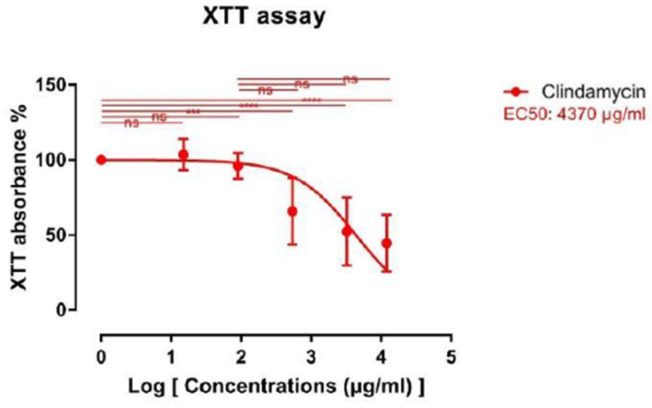
Sigmoidal dose-response curve for XTT assay. Data are shown as mean ± standard deviation. Asterix denotes statistically significant difference vs. the control group (ns = not significant; *** = *p* < 0.001; **** =*p* < 0.0001). ns means non-significant. EC50 characterizes the concentration leading to a 50% reduction.

Results of the -(4,5-Dimethylthiazol-2-yl)-2,5-diphenyltetrazoliumbromid (XTT) assay are depicted in Figure 3. Clindamycin in the concentrations of 15 and 90 μg/mL did not lead to any significant differences compared to the control. Absorbance levels of samples treated with 540 μg/mL clindamycin were 65.84 ± 22.19%. This decrease was statistically significant when compared to the control (*p* < 0.001). For 3240 and 12,000 μg/mL, absorbance was 52.38 ± 22.72 and 44.7 ± 18.89%, respectively. Again, when compared with the control group, reduction of cell viability was statistically significant. Although a trend toward a dose-dependent reduction of absorbance was observed, differences between study groups did not reach statistical significance.

The XTT assay for cefazolin treated adipocytes did not result in interpretable data as the XTT solution cross-reacted with the cefazolin, and thus, no data are presented.

### 2.5. G3PDH Assay

Lipid metabolism was significantly impaired starting at concentrations of 540 μg/mL clindamycin (49.67 ± 22.62%, *p* < 0.0001) compared to plain saline (0.0 μg/mL). Similar to the cell viability tests, higher concentrations of clindamycin led to a stronger decrease of lipid metabolism (AB4: 25.33 ± 15.71% and AB5: 14.47 ± 14.73%, *p* < 0.0001). We did not observe significant differences between the study groups themselves.

**Figure 4:**
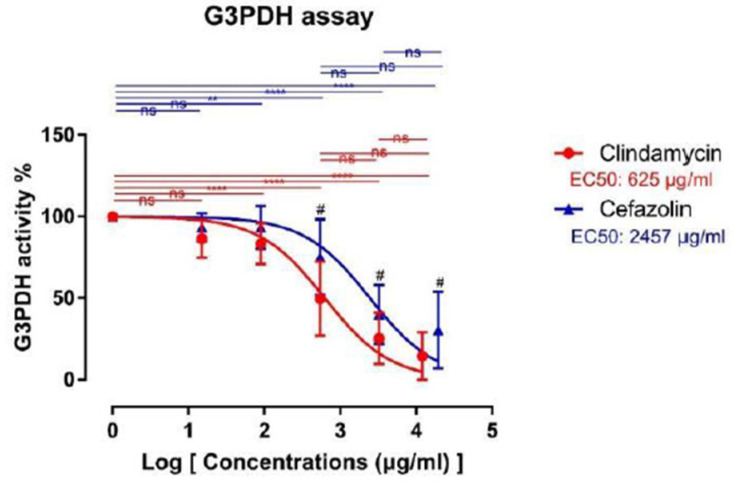
Sigmoidal dose-response curve for G3PDH assay. Data are shown as mean ± standard deviation. Asterix denotes statistically significant difference vs. the control group (ns = not significant; ** = *p* < 0.01; **** = *p* < 0.0001). Brackets indicate statistically significant differences between corresponding groups of clindamycin and cefazolin treated adipocytes (# *p* = <0.05). ns means non-significant. EC50 characterizes the concentration leading to a 50% reduction.

Cefazolin had a comparable impact on adipocytes with 15 and 90 μg/mL not leading to significant alterations of lipid metabolism. When compared to the control, 540, 3250 and 19,440 μg/mL cefazolin significantly reduced the measured absorbance during the G3PDH assay, which translates to significantly reduced adipocyte lipid metabolism (Control vs. AB3: 75.17 ± 23.29, *p* < 0.01; vs. AB4: 40.01 ± 18.08, and AB5: 30.43 ± 23.56%, *p* < 0.0001). Again, inter-group comparisons did not yield significant differences between study groups. Similar to the live/dead assay, comparisons between corresponding clindamycin and cefazolin groups showed significantly stronger reductions of G3PDH activity after clindamycin treatment for groups AB3, AB4 and AB5. The sigmoidal dose-response curve for G3PDH is shown in Figure 4.

### 2.6. Reactive Oxygen Species (ROS) Assay

Reactive oxygen species production in adipocytes incubated with clindamycin progressively increased starting from 90 μg/mL to 3240 μg/mL compared to the control group (AB2: 162.8 ± 43.67, *p* < 0.001; vs. AB3: 288.9 ± 140.6, and AB4: 391.9 ± 201.9%, *p* < 0.0001). Interestingly, at 12,000 μg/mL clindamycin (AB5), we observed a decrease of ROS compared to group AB4, yet, with significant higher values compared to the control (AB5: 238.9 ± 202.9%, *p* < 0.01). Statistically significant differences between study groups and between group AB1 and the control were not observed.

**Figure 5:**
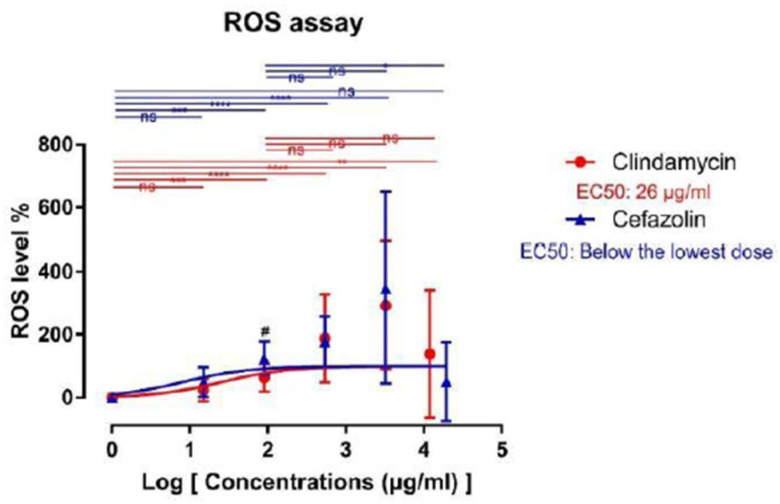
Sigmoidal dose-response curve for ROS assay. Data are shown as mean ± standard deviation. Asterix denotes statistically significant difference vs. the control group (ns = not significant; ** = *p* < 0.01, *** = *p* < 0.001, **** = *p* < 0.0001). Brackets indicate statistically significant differences between corresponding groups of clindamycin and cefazolin treated adipocytes (# = *p* < 0.05). ns means non-significant. EC50 characterizes the concentration leading to a 50% increase.

In accordance with the increase of ROS after clindamycin treatment, cefazolin led to a very similar increase of oxidative stress on adipocytes. ROS occurrence was significantly higher for group AB2 with a gradual increase for AB3 and AB4 (Control vs. AB2: 225.1 ± 53.3%, *p* < 0.001; vs. AB3: 278.1 ± 79.65, and AB4: 447.7 ± 303%, *p* < 0.0001). Again, the highest concentration of cefazolin (AB5: 150.7 ± 125.9%) showed a paradox decrease when compared to group AB4 and was not significantly different when compared to the control. Of note, absorbance values of samples treated with 90 μg/mL cefazolin were significantly higher than samples treated with the highest concentration of 19,440 μg/mL (AB2: 225.1 ± 53.3% vs. AB5: 150.7 ± 125.9%, *p* < 0.05). In the comparison of clindamycin and cefazolin treated groups, we found a statistically significant difference between both groups at 90 μg/mL (62.77 ± 43.67% vs. 25.13 ± 53.3%, *p* < 0.05). Data regarding the ROS assay is displayed in Figure 5.

### 2.7. Morphologic Assessment of ADSC

**Figure 6:**
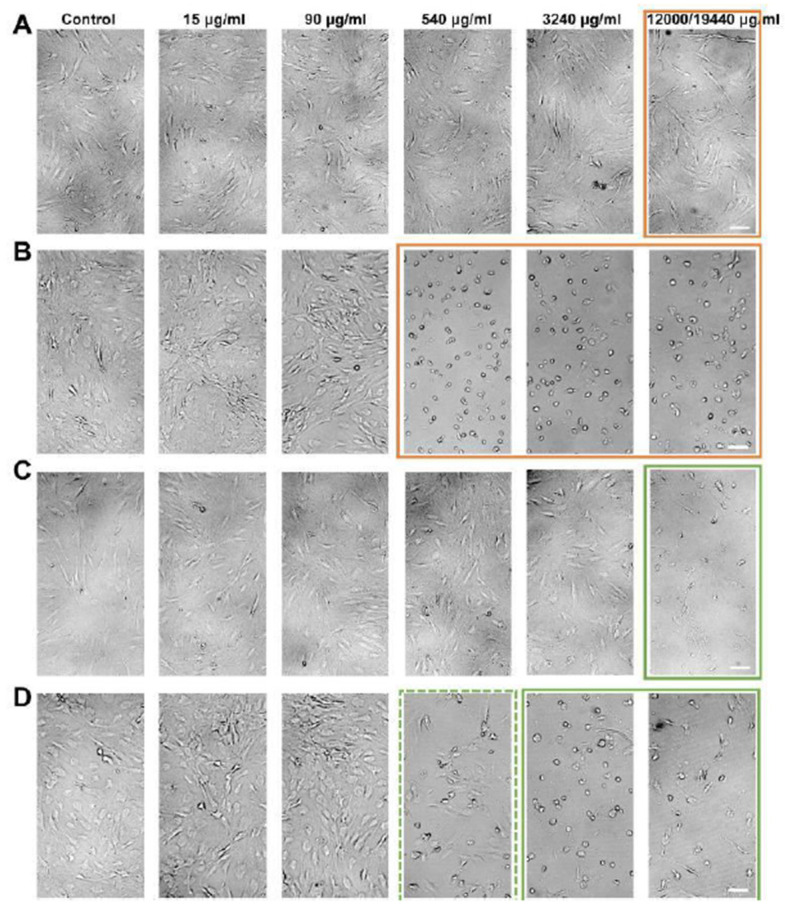
Example pictures of morphological changes in ADSC at 5× magnification. (**A**,**B**) ADSCs treated with clindamycin for 24 h or 9 days, respectively. The orange frame marks the reduced cell density (**A**) or the loss of fibroblast-like morphology (**B**). (**C**,**D**) ADSCs treated with cefazolin for 24 h or 9 days, respectively. The green frame marks reduced cell density and loss of fibroblast-like morphology.

Figure 6 shows an exemplary presentation of morphological changes in ADSCs after clindamycin and cefazolin incubation. For clindamycin, we observed a decreased cell density after 24 h incubation at a concentration of 12,000 μg/mL while maintaining their fibroblast-like morphology. Following nine days of incubation, ADSCs lost their typical morphology starting at a concentration 540 μg/mL, with cells becoming round. Additionally, cell density gradually decreased.

Cefazolin incubation showed a loss of fibroblast-like morphology in almost all cells after 24 h incubation with the highest concentration (19,440 μg/mL). When incubating ADSCs for nine days, all ADSC became spherical with complete absence of ADSCs proliferation and reduced densities in groups AB4 and AB5.

### 2.8. Analysis of ADSC Differentiation Potential

**Figure 7:**
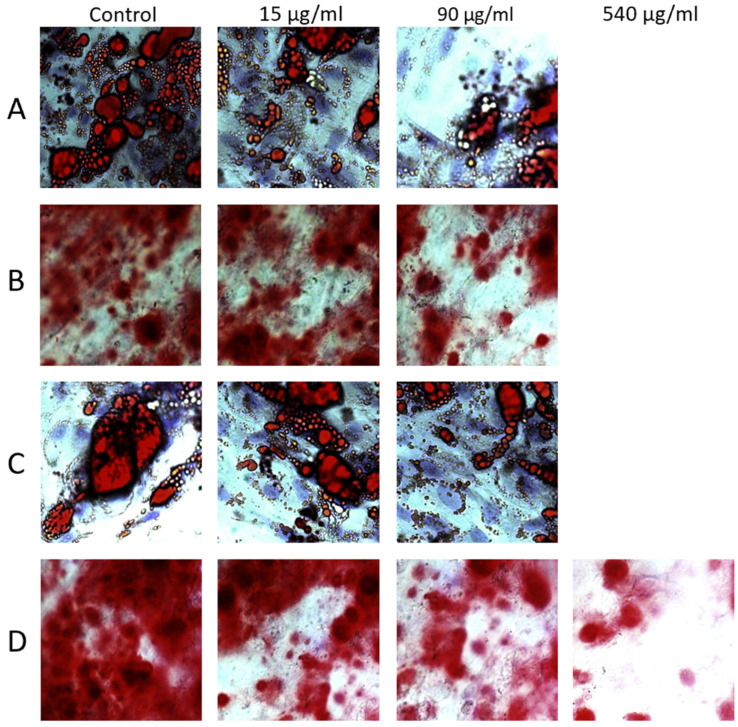
Example pictures of adipogenic and osteogenic differentiation of ADSCs at 5× magnification. Oil Red O (**A**,**C**) and Alizarin Red (**B**,**D**) analysis of differentiation potential for ADSCs treated with clindamycin (**A**,**B**) and cefazolin (**C**,**D**). ADSCs were not able to differentiate into adipocytes after treatment with concentrations higher than 90 μg/mL for both antibiotics, and into osteoblasts after incubation with concentrations higher than 90 μg/mL clindamycin and 540 μg/mL cefazolin.

**Figure 8:**
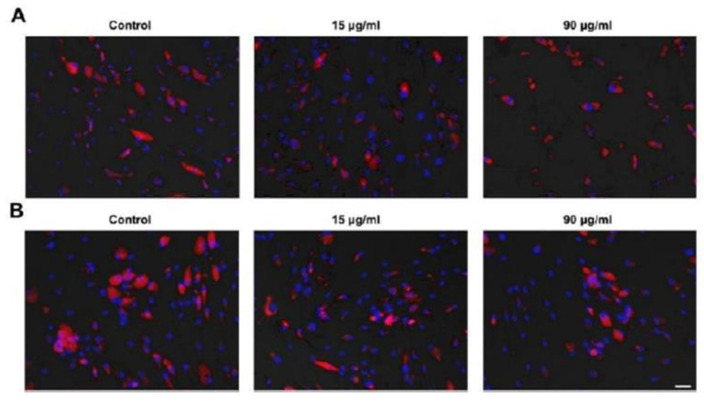
Example pictures of reduced differentiation potential of ADSCs at 5× magnification. ADSCs treated with clindamycin (**A**) and cefazolin (**B**). Differentiated adipocytes appear with a co-staining of red lipid droplets and blue cell nuclei. ADSCs appear with only blue cell nuclei without red lipid droplets. In both groups, adipogenic differentiation capability was reduced through antibiotic treatment.

Incubation of ADSCs with both clindamycin and cefazolin, inhibited the adipogenic capacity at concentrations higher than 90 μg/mL.In both study arms, ADSCs in groups AB3, AB4, and AB5 did not differentiate into adipocytes at all during the adipogenic differentiation protocol. Stem cells treated with 15 and 90 μg/mL, however, still successfully differentiated, with a trend toward weaker differentiation for higher antibiotic concentrations shown with Oil Red O and fluorescent staining (Figure 7 and Figure 8). For osteogenic differentiation we observed similar results. ADSCs were not able to differentiate into osteoblasts after incubation with concentrations higher than 90 μg/mL clindamycin and 540 μg/mL cefazolin as shown by Alizarin Red staining. Again, we found a trend toward weaker differentiation for higher concentration (Figure 7).

### 2.9. Antibiotic Susceptibility Test

A dose of 2 μg clindamycin resulted in an inhibition zone with a diameter of 28 mm which can be considered susceptible according to the criteria of Clinical and Laboratory Standards Institute (CLSI) (susceptible diameter range: 24–30 mm). The converted Minimum Inhibitory Concentration (MIC) for clindamycin in this experiment was ≤0.5 μg/mL, accordingly.

Similarly, 30 μg of cefazolin produced an inhibitory zone with a diameter of 33 mm (susceptible diameter range: 29–35 mm, according to the CLSI standard). Thus, the MIC for cefazolin in this experiment was ≤8 μg/mL.

## 3. Discussion

To the best of our knowledge, this is the first in vitro study analyzing the effects of the antibiotics clindamycin and cefazolin at different concentrations in fat grafting. We found that both antibiotics led to a significant, dose-dependent impairment of cell viability, lipid metabolism, regeneration potential, and ROS pathways in adipocytes and adipose-derived stem cells, the most important cell types to promote and maintain fat graft survival. Although low concentrations of clindamycin and cefazolin displayed considerable negative effects, the lowest concentrations have been found to be still effective in preventing bacterial growth.

We applied the paper disk diffusion test, which is the most widely used method in clinical microbiology testing of antibiotics, to predict the inhibitory efficacy of very low concentrations of the two antibiotic solutions. This test offers the advantages of ease of use, reproducibility, visualization of results, and standardization [19,20]. According to the CLSI guidelines, this paper disk diffusion test is suitable for fast growing bacteria, such as staphylococcus aureus, one of the main pathogens in postoperative infections after lipofilling procedures [21]. Applying this standardized test, we found a minimum inhibitory concentration of ≤0.5 μg/mL for clindamycin and ≤8 μg/mL for cefazolin in our experimental setting. Clindamycin at a concentration of only 90 μg/mL had significant negative effects on adipocyte viability, whereas obvious toxicity of cefazolin was observed starting from a higher concentration of 540 μg/mL. At the highest concentration, cell viability of adipocytes remained at approximately 50% compared to the control after cefazolin treatment while almost no surviving adipocytes could be found in the corresponding clindamycin group. In regard to lipid metabolism, both antibiotics led to a significant reduction at concentrations from 540 μg/mL onwards. However, the calculated EC50 values of clindamycin were considerably lower compared to those calculated for cefazolin. Therefore, our results indicate that clindamycin is more cytotoxic for adipogenic cells compared to cefazolin. These findings are well in line with a study by Duewelhenke et al., analyzing the effect of clindamycin and cefazolin, among others, on osteoblasts and epithelial cells [22]. The authors demonstrated that common antibiotics in concentrations which are regularly reached in vivo during clinical application, can be cytotoxic and/or cytostatic for osteoblasts and epithelial cells. This was analyzed by means of cell death quantification, proliferation assay, metabolic activity, and lactate production. In accordance with our study results, Duewelhenke et al. found considerably lower 20% and 50% inhibitory concentrations for clindamycin compared to those of cefazolin [22]. In the present study, analysis of adipocytes and ADSCs viability, metabolism and ROS accumulation also resulted in lower inhibitory doses for clindamycin compared to cefazolin. One possible cause could lie in the different pharmacological properties of clindamycin and cefazolin. While cefazolin is a hydrophilic molecule, clindamycin is a lipophilic drug [23,24]. The latter is more readily distributed into adipose tissue and therefore can reach higher tissue concentrations and distribution compared to less lipophilic drugs. On the contrary, studies have shown that the cefazolin concentrations in adipose tissue are 30 times lower than corresponding serum concentrations, indicating that the high-water solubility of cefazolin causes the weak distribution in adipose tissue [25]. It can be argued that the tendency of clindamycin to enrich in adipose tissue is a significant factor in the mediation of the cytotoxicity in our experimental setting. This might indeed play an important role in the clinical application during lipofilling procedures, when fat grafts are rinsed with antibiotic solutions prior to transplantation. Here, a more lipophilic drug might accumulate more within the fat tissue and more residual antibiotics might be left within the fat graft during transplantation. In our experimental setting; however, we directly incubated isolated adipocytes and ADSCs with clindamycin and cefazolin, and thus, the lipophilic or hydrophilic properties should not play a substantial role as cells had sufficient contact with both antibiotic solutions during the incubation steps. Therefore, the higher cytotoxic and inhibitory effect of clindamycin compared to cefazolin cannot only be based on its lipophilic characteristics. This is supported by the fact that Duewelhenke et al. found similar results for osteoblast and epithelial cell lines [22]. To date, the specific pathways responsible for the cytotoxic effects of the antibiotics are not fully understood. In the present study, we found significantly increased ROS levels in adipocytes treated with 90 μg/mL clindamycin or cefazolin. Under normal conditions, ROS are a regular product of the cell metabolism, yet, if the metabolism is disturbed, ROS accumulation can cause damage to crucial cell structures, ultimately leading to cell death [26]. Interestingly, adipocyte viability started to be significantly reduced at 90 μg/mL of clindamycin and 540 μg/mL of cefazolin, implying that there may be additional pathways to damage adipocytes, such as interference with lipid droplets by fat-soluble clindamycin. The paradoxical decrease of ROS levels at the highest antibiotic concentrations, initially raised questions, might be explained, however, by a rapid death of large populations of adipocytes within a short time period, leading to a lack of ROS production by surviving cells [27].

Overall, we found that clindamycin has a higher toxicity toward adipocytes and ADSCs compared to cefazolin. However, both antibiotics, while being effective in preventing bacterial growth at very low concentrations, had impactful negative effects on cell viability and metabolism at barely higher concentrations. Although clinical trials are without doubt necessary to confirm these effects in a clinical setting, the results of this study can be used to translate these experimental data and to propose safe and effective concentrations of clindamycin and cefazolin for the prevention of pathogen-related infections after lipofilling procedures.

The presented study has some limitations which need to be discussed.

As this was the first approach to investigating the negative effects of clindamycin and cefazolin in the setting of fat grafting, we initially performed an in vitro study. However, the results of these experiments may not directly translate to clinical conditions, as the human body has a more complex regulatory system [28]. During lipofilling procedures, the fat graft is rinsed with antibiotic solutions and subsequently injected into the desired body area. For the assessment of adipocyte viability and ADSCs proliferation and differentiation capacity, it was necessary to wash the fat tissue with PBS after antibiotic rinsing during the isolation process. This would lead to a false negative result, due to the removal of antibiotic residue. Therefore, we had to adjust the study design and directly incubate the isolated adipocytes and ADSCs with the antibiotics. The gathered data have to be treated with caution as direct incubation with antibiotics may lead to a stronger cytotoxicity as it would be in an in vivo model. This limitation is aggravated if the potential dilution or inactivation of antibiotic solutions through serum and tissue proteins and fluids is taken into consideration. The results need to be further validated in in vivo models and ultimately, clinical trials.

Another limitation is the restriction of two antibiotic solutions used in the presented study. We chose the most common antibiotics used to prevent lipofilling-related infections, namely clindamycin and cefazolin, for our experiments. Yet, a vast number of alternative drugs exist, some of which without proven cytotoxicity [22]. Different antibiotics might be more suitable for lipofilling procedures, but basic research needs to be conducted beforehand.

## 4. Materials and Methods

### 4.1. Study Design

This study protocol was approved by the local Ethics Committee (Processing number: 837.436.16 (10755), 12 May 2017, Mainz, Germany). Excessive fat tissue was harvested from 24 healthy female patients during regular lipofilling procedures to the breast. Attained lipoaspirate was split to obtain adipocytes and ADSCs on the one hand, and for antibiotic susceptibility testing on the other hand. Isolated cells were co-incubated with six different concentrations of clindamycin and cefazolin and subjected to a series of assays to evaluate cell viability, lipid metabolism, and stem cell capacities. Antibiotic concentrations were chosen based on available data on their impact on different cell lines and the manufacturers’ recommended dilution [22,29]. Experimental concentrations and study groups are shown in Table 1.

### 4.2. Lipoaspirate Harvesting and Sample Processing

Informed consent was gathered preoperatively from every study patient. Adipose tissue was harvested during water-jet assisted liposuction using the body-jet^®^ evo system (Human Med^®^, Schwerin, Germany) in the LipoCollection mode under general anesthesia. Donor sites were the upper thigh or the abdomen, depending on the fat distribution of each individual patient. We used a tumescent solution consisting of 0.5 mg Epinephrine (Suprarenin^®^ 1 mg/mL, Sanofi, Paris, France) diluted in 1 L of Ringer’s solution. After injection and distribution of the tumescent solution in the corresponding body areas in multilayer/multichannel, liposuction was performed with 4 mm cannulas (Ponsamed, Bonn, Germany) under −350 mmHg and adipose tissue was collected in a LipoCollector. Subsequently, the aqueous phase was drained and the adipose tissue transferred to the laboratory for further processing and experiments, respectively.

### 4.3. Sample Processing

Immediately after harvesting adipose tissue, samples were transferred to a sterile bench to ensure its vitality. Fat tissue was rinsed multiple times with phosphate-buffered saline (PBS, Invitrogen, Carlsbad, CA, USA) and excess blood, tumescent solution, and cell debris was removed by centrifugation at 6000 rpm for 5 min. and disposal of the supernatant. Then, 25 mL of adipose tissue was mixed with 50 mL of 0.1% type I collagenase in phosphate-buffered saline and incubated for 30 min under continuous shaking in a water bath at 37 °C. To stop the enzymatic reaction, culture medium (DMEM High Glucose +10% FBS +1% penicillin-streptomycin) was added in a ratio of 1.5:1. After centrifugation at 1500 rpm for 5 min, the tissue sample appeared in three layers, with the top layer consisting of mainly adipocytes, the middle layer consisting of liquid, and the bottom layer being the stromal vascular fraction (SVF) containing ADSCs. The top and bottom layer were carefully removed, filtered through a 300 μm cell strainer to remove debris and clumps, and used for the subsequent analysis. ADSCs were cultivated until passage 3 as previously described [30].

### 4.4. Characterization of ADSCs into Osteogenic and Adipogenic Lineages

ADSCs were characterized by adipogenic and osteogenic differentiation as well as Fluorescence-Activated Cell Sorting (FACS) as described in our previous study [31]. Briefly, for adipogenic differentiation, we used an induction medium containing 500 µM 3-Isobutyl-1-methylxanthin (IBMX), 1 µM dexamethasone and 1 µM indomethacin (incubation for 2 days) and the preservation medium containing 10 µg/mL insulin (incubation for 1 day), which were alternately changed eight times. All solutions were obtained from Sigma-Aldrich, Darmstadt, Germany. Successful differentiation was detected by Oil Red O staining of lipid vacuoles within adipocytes. Osteogenic differentiation was achieved by incubation with a medium containing 50 µM L-ascorbat-2-phosphate, 0.1 µM dexamethasone and 10 mM beta-glycerophosphate disodium for 14 days and subsequent Alizarin Red staining of osteoclasts. Through FACS sorting, we analyzed the representation of the surface markers CD29, CD31, CD45, CD73 and CD90 on cultivated ADSCs in passage three.

### 4.5. Hoechst/Propidium Iodide (PI) Fluorescence Staining

Live/Dead fluorescence staining was performed to differentiate vital adipocytes from dead cells after antibiotic incubation with several concentrations (Table 1). For this purpose, 100 µL of purified adipocytes were transferred in 96-well plates and cultured at 37 °C, 5% CO_2_ with 100 µL of cell culture medium containing antibiotic supplements according to Table 1. After 24 h, wells were stained with Hoechst 33342 and propidium iodide for 15 min. Cells were then transferred to a hemocytometer and counted under the microscope by selecting three random areas. Live adipocytes appeared as cells with blue-stained nuclei with lipid droplets and dead adipocytes with red-stained nuclei. The percentage of survived cells was expressed as (viable adipocytes)/(total adipocytes) ratio and is given as mean ± standard deviation.

### 4.6. XTT Assay

Adipocyte viability was analyzed by means of XTT assay kit (Sigma-Aldrich, Germany). In 24-well plates, 200 µL adipocytes were mixed with 1.4 mL cell culture medium containing the antibiotics and incubated for 24 h. XTT labeling reagent and electron coupling reagent were added in a 50:1 ratio (800 µL) and samples were incubated for four hours, according to the manufacturers’ recommendations. Subsequently, absorbance was measured at 450 nm using an ELISA reader (Multiskan SkyHigh, ThermoFisher Scientific, Waltham, MA, USA) with a reference wavelength of 650 nm. Absorbance values of the corresponding control group (0.0 µg/mL antibiotics) are considered to be 100%. Results are expressed as absorbance of the test ample in relation to the control and is given as mean ± standard deviation.

### 4.7. Glycerol 3-Phosphate Dehydrogenase (G3PDH) Assay

Adipocyte lipid metabolism was analyzed by G3PDH assay (Sigma-Aldrich, Darmstadt, Germany). 100 µL adipocytes were incubated with 100 µL cell culture medium with antibiotics for 24 h. Then, cells were ruptured by repeated freezing through liquid nitrogen and thawing. After centrifugation at 13000 rpm for 10 min., 5 μL of the middle layer were diluted in 20 μL of G3PDH assay buffer, 1 μL G3PDH probe and 1 μL G3PDH substrate. Absorbance was measured at a wavelength of 450 nm once per minute for 30 min. G3PDH activity is proportional to the absorbance increase per time and was calculated as follows:

G3PDH activity=(ODT2−ODT1)/(T2−T1)

### 4.8. Reactive Oxygen Species (ROS) Assay

ROS were analyzed by means of ROS assay kit (Promega, Fitchburg, MA, USA) according to the manufacturer’s recommendations. Briefly, after incubation with different concentrations of antibiotics, adipocytes were transferred to 96-well plates and H2O2 substrate solution was added. After 6 h of incubation, 50 μL of the sample solution were mixed with 50 μL of ROS detection solution and incubated for 20 min. ROS were quantified using a luminescent reader (GloMax, Promega, Madison, WI, USA).

### 4.9. Morphologic Assessment of ADSCs

Morphologic alterations of ADSCs through antibiotics were monitored by light microscopy. ADSCs of passage 5 were incubated with cell culture medium containing different doses of antibiotics according to Table 1. Morphologic changes were observed under a light-microscope at 5× magnification at 24 h and 9 days. Loss of stem cell shape was defined as loss of fibroblast-like appearance with irregular or increasing round shape and less occurrence of dividing cells.

### 4.10. Analysis of ADSC Differentiation Potential

Adipogenic and osteogenic differentiation was performed as described above.

Differentiation into adipocytes was detected by Oil Red O staining of lipid vacuoles. Osteogenic differentiation, on the other hand, was analyzed by means of Alizarin Red staining of osteoclasts.

Due to the fact that differentiation of ADSCs into adipocytes is crucial for maintain fat graft survival, we additionally distinguished differentiated adipocytes from not-differentiated ADSCs with fluorescence staining using Nile Red and Hoechst 33342. Cells were thoroughly washed and fixed with 4% formaldehyde. After rinsing with PBS, staining was performed with Nile Red (10 µg/mL) and nuclei were counterstained with Hoechst 33342 (1 µg/mL), leading to lipid droplets appearing with red fluorescence and nuclei with blue fluorescence. Differentiated adipocytes were co-stained with both Nile Red and Hoechst 33342.

### 4.11. Antibiotic Susceptibility Test

To identify the minimal inhibitory dose of clindamycin and cefazolin in fat grafting, 5 mL of aspirated fat from each patient was mixed with 5 mL of a standard laboratory testing control strain of Staphylococcus aureus containing approximately 5 × 10^3^ colony forming units (Staphylococcus aureus ATCC 25923) [32]. A sterile cotton swab was dipped into the mixture and transferred to Mueller Hinton agar plates and evenly distributed around the whole surface. Bacterial incubation of agar plates was performed at 37 °C for 36 h. Subsequently, paper disks were loaded with different doses of clindamycin and cefazolin (2, 15, 30 and 45 yg) and placed on the agar plates. Following a second incubation period of 18 h, the diameter of the inhibition zone around the paper plates containing antibiotics was measured under light microscopy at 3× magnification. The Minimum Inhibitory Concentration (MIC) was calculated based on the CLSI guidelines [33].

### 4.12. Statistics

Acquired data were tested for statistically significant differences with GraphPad prism 7 (GraphPad Software, Boston, MA, USA). All data were tested for normal distribution by means of the D’Agostino and Pearson normality test. Subsequently, parametric tests were used for normally distributed data, and non-parametric tests were used for non-normally distributed data. Parametric one-way ANOVA with Tukey’s multiple comparison post-hoc test was used for live/dead cell analysis of clindamycin treated adipocytes. For the remaining assays, the non-parametric Kruskal-Wallis test followed by Dunn’s multiple comparison test was applied. All data are expressed as mean ± standard deviation (SD) and significance level was set to *p* < 0.05.

## 5. Conclusions

This study shows that clindamycin as well as cefazolin have a cytotoxic effect on fat grafts at relatively low concentrations, leading to impaired cell viability, metabolism, and adipogenesis. Proposed safe concentrations in the chosen experimental setting were 15 μg/mL for clindamycin and 90 μg/mL for cefazolin. However, these low concentrations are still effective in inhibiting bacterial growth in vitro.

## Figures and Tables

**Table 1:** Experimental concentrations of clindamycin phosphate and cefazolin.

Group	Control	AB1	AB2	AB3	AB4	AB5
Clindamycin phosphate (µg/mL)	0	15	90	540	3240	12,000
Cefazolin (µg/mL)	0	15	90	540	3240	19,440

## Data Availability

The data presented in this study are available on request from the corresponding author.
